# Progression of pulmonary hyperinflation and trapped gas associated with genetic and environmental factors in children with cystic fibrosis

**DOI:** 10.1186/1465-9921-7-138

**Published:** 2006-11-30

**Authors:** Richard Kraemer, David N Baldwin, Roland A Ammann, Urs Frey, Sabina Gallati

**Affiliations:** 1 Department of Paediatrics, University of Berne, Inselspital CH-3010 Berne, Switzerland.; 2 Division of Pediatric Respiratory Medicine, Department of Pediatrics, University of Berne, Inselspital, CH-3010 Berne, Switzerland.; 3 Division of Human Genetics, Department of Pediatrics, University of Berne,, Inselspital, CH-3010 Berne, Switzerland.

## Abstract

**Background:**

Functional deterioration in cystic fibrosis (CF) may be reflected by increasing bronchial obstruction and, as recently shown, by ventilation inhomogeneities. This study investigated which physiological factors (airway obstruction, ventilation inhomogeneities, pulmonary hyperinflation, development of trapped gas) best express the decline in lung function, and what role specific *CFTR *genotypes and different types of bronchial infection may have upon this process.

**Methods:**

Serial annual lung function tests, performed in 152 children (77 males; 75 females) with CF (age range: 6–18 y) provided data pertaining to functional residual capacity (FRC_pleth_, FRC_MBNW_), volume of trapped gas (V_TG_), effective specific airway resistance (sR_eff_), lung clearance index (LCI), and forced expiratory indices (FVC, FEV_1_, FEF_50_).

**Results:**

All lung function parameters showed progression with age. Pulmonary hyperinflation (FRC_pleth _> 2SDS) was already present in 39% of patients at age 6–8 yrs, increasing to 67% at age 18 yrs. The proportion of patients with V_TG _> 2SDS increased from 15% to 54% during this period. Children with severe pulmonary hyperinflation and trapped gas at age 6–8 yrs showed the most pronounced disease progression over time. Age related tracking of lung function parameters commences early in life, and is significantly influenced by specific *CFTR *genotypes. The group with chronic *P. aeruginosa *infection demonstrated most rapid progression in all lung function parameters, whilst those with chronic *S. aureus *infection had the slowest rate of progression. LCI, measured as an index of ventilation inhomogeneities was the most sensitive discriminator between the 3 types of infection examined (*p *< 0.0001).

**Conclusion:**

The relationships between lung function indices, *CFTR *genotypes and infective organisms observed in this study suggest that measurement of other lung function parameters, in addition to spirometry alone, may provide important information about disease progression in CF.

## Background

Cystic fibrosis (CF) is the most common life-shortening genetic disease among Caucasians, being caused by mutations of the cystic fibrosis transmembrane conductance regulator (*CFTR*) gene [1]. Dysregulation of epithelial chloride channels results in dehydration of the luminal surface of exocrine cells, increased mucus viscosity and altered mucociliary clearance. The occurrence of these changes most likely follows periciliary liquid layer depletion and together are responsible for the CF phenotype [2-5]. Recent data have linked the abnormal ion transport properties of CF airway epithelia to depleted airway surface liquid volume, reflecting the combined defects of accelerated Na^+ ^transport and the failure to secrete Cl^-^. Depletion of a specific compartment of the airway surface liquid, i.e. the periciliary fluid, appears to abrogate both cilia-dependent and cough clearance [4,6]. Mucus clearance is a major component of the lung's innate defense mechanism. The efficiency of mucus clearance reflects in part the volume of airway surface liquid (ASL) on airway surfaces. The ASL is comprised of a periciliary liquid layer (PCL), which lubricates the cell surface, and a mucus layer, which traps airborne particles and pathogens [7]. Cystic fibrosis airways exhibit Na^+ ^hyperabsorption and Cl^- ^hyposecretion, which leads to ASL volume depletion, mucus stasis, and mucus plugging. These mucus plugs are the site of persistent bacterial infections that lead to a massive neutrophil infux and raised immune responses that promote airway remodeling [8]. Regulation of ASL volume is poorly understood [9], although Tarran et al. recently showed that CF airway epithelia lack *CFTR*-dependent Cl^- ^secretion and exhibit Na^+ ^hyperabsorption, leaving CF cultures only partially able to adjust ASL volume [9,10]. Bacterial colonization, infection, and chronic pulmonary inflammation develop subsequently. Pulmonary complications account for most of the morbidity and mortality in CF patients, and the majority of patients with CF die from respiratory failure due to endobronchial infection and neutrophil-dominated inflammation [11,12]. Advances in the care of patients with CF have improved survival, and as a result, patients with the disease now often live beyond the third decade [13]. The heterogeneous course of disease progression observed in CF remains incompletely explained and most likely reflects the influence of multiple, interrelated factors. These may include differences in *CFTR *mutation and presence of infective organisms within the respiratory tract [14,15].

Previous studies in patients with CF have demonstrated the presence of ventilation inhomogeneities [16,17], pulmonary hyperinflation [18-21] and gas trapping [22] as early as during the first years of life [17-19], and these may progress during childhood [16,20]. Only few observational population-based studies [23-26] have specifically evaluated progression of lung function characteristics such as airway obstruction, ventilation inhomogeneities, pulmonary hyperinflation, and development of trapped gas over time. We have previously reported observations that inequalities in ventilation occur significantly earlier in the course of lung function decline than other functional characteristics [16]. Here we hypothesize that functional consequences of lung disease in CF extend beyond simple bronchial obstruction, and should be examined in terms of alveolar volume, including gas trapping, as well as alveolar ventilation.

In the current study we investigated (*i*) whether or not pulmonary hyperinflation and/or trapped gas represent further indicators of functional deterioration that should be monitored during childhood. Moreover, we attempted (*ii*) to define the role of specific *CFTR *genotypes and the influence of *PA *infection upon rates of disease progression. Finally, we intended (*iii*) to demonstrate whether or not those young children in whom respiratory dysfunction occurs earliest and with greatest severity, are more likely to follow a more rapid decline in pulmonary function, consistent with the concept of functional tracking over time. Progressive functional deterioration of this type has been previously reported in several chronic respiratory diseases including bronchial asthma [27,28] chronic lung disease of infancy [29] and cystic fibrosis [16,25,26,30].

## Study population and methods

### Bernese Cystic Fibrosis Patient Data Registry

This prospective registry was initiated in 1978 as an extension of the American Cystic Fibrosis Patient Registry founded by Warwick in 1966 [31], and comprises systematic clinical and lung function data obtained from CF patients reviewed as inpatients and outpatients over a time span of 28 years. This comprehensive source provided data for the observational cohort study which were reviewed according to the following inclusion criteria: (*i*) CF diagnosis based on the presence of characteristic phenotypic features [32,33], (*ii*) confirmed by a duplicate quantitative pilocarpine iontophoresis sweat test measuring both Na and Cl values > 60 mEq/L as well as by (*iii*) genotype identification using extended mutation screening of both alleles [34,35], and (*iv*) complete documentation of a minimum of 4 lung function tests performed annually between age 6–18 y. The study protocol was approved by the Departmental Ethics Committee of the University Children's Hospital and by the Government Ethics Committee of the State of Berne, Switzerland. Parts of the lung function data from this cohort have been reported previously [16].

### Pulmonary Function Measurements

Spirometry and flow volume curves were obtained by whole body plethysmography using a volume-constant, pressure-variable plethysmograph with air bag body temperature and pressure saturated (BTPS) compensation unit (BodyScreen, Jaeger Würzburg, Germany) until December 4, 1993. Thereafter, the MasterLab plethysmograph was employed (MasterLab, Jaeger Würzburg, Germany). This instrument uses electronic BTPS-compensation. Children were requested to breathe at coached normal frequency during shutter closure (no panting) for measurements of functional residual capacity (FRC_pleth_). Prior to plethysmographic measurements, resting end-expiratory lung volume (FRC_MBNW_) and quantification of ventilation inhomogeneities (LCI) was determined by open-circuit multibreath nitrogen washout (MBNW) technique [36] using the Pediatric Pulmonary Unit (SensorMedics 2200, Yorba Linda, Ca, USA). This procedure enabled longitudinal assessment of the following parameters: (*i*) FRC_pleth _measured by whole-body plethysmography, (*ii*) FRC_MBNW _measured by MBNW technique, combining both measurements to calculate (*iii*) an index of the volume of trapped gas (V_TG _= FRC_pleth _-FRC_MBNW_) [37] and (*iv*) effective specific airway resistance (sR_eff_). Following a short rest period, indices of forced expiratory air flow limitation including (*v*) forced vital capacity (FVC), (*vi*) forced expired volume in one second (FEV_1_) and (*vii*) maximal expired volume at 50 percent of FVC (FEF_50_) were calculated from maximal expiratory flow volume curves. All measurements were stored for offline analysis and the 3–5 technically most satisfactory maneuvers were chosen for analysis using a computer system adapted for children (MasterLab, Jaeger Würzburg, Germany). With the exception of LCI, all values were expressed as a standard deviation score (SDS) based on gender- and age-specific regression equations [38-40]. Interpretation of LCI data required a z-transformation of log-transformed gender-specific data obtained in healthy subjects [38]. Technical details are given elsewhere [16,41].

We have recently identified lung clearance index (LCI) obtained by multiple breath nitrogen washout (MBNW) technique [16], followed by MEF_50 _and FRC_pleth _as the strongest indicators of disease progression. Furthermore, LCI was observed to reflect progressive deterioration in lung function earlier in life than alterations occurring in FEV_1. _Assessment of the degree of airway obstruction alone may therefore be inadequate for following progression of lung disease in CF. For example, in patients with end-stage CF lung disease, pulmonary hyperinflation is correlated with gas exchange characteristics [42]. Physiologically, at least five potential mechanisms of functional deterioration exist that may alter gas exchange, including: (*a*) progression of pulmonary hyperinflation, represented by FRC_pleth_, (*b*) progression of ventilation inhomogeneities (LCI), (*c*) development of trapped gas (V_TG_), (*d*) airway narrowing (sR_eff_) and (*e*) small airway disease (FEV_1 _and FEF_50_).

Therefore, progression of disease as quantified by the tracking of lung function decline was evaluated by stratification of patients into 4 subgroups according to the following criteria:

1) Group FN included 24 patients with functionally normal FRC_pleth _and normal LCI at entry.

2) Group VIH comprised 71 patients in whom only ventilation inhomogeneities were present (normal FRC_pleth_; LCI > 2SDS; no trapped gas).

3) Group PH included 29 patients with pulmonary hyperinflation (FRC_pleth _> 2SDS) in the absence of trapped gas. Each of these children also had ventilation inhomogeneities present (LCI > 2SDS).

4) Group PH&TG comprised 28 patients with pulmonary hyperinflation (FRC_pleth _> 2SDS), trapped gas (V_TG _> 2SDS) and elevated LCI.

### Genotype analysis

Genomic DNA was extracted from EDTA blood samples using the QIAamp Maxi Kit (Qiagen) according to the manufacturer's recommendations and quantified by spectrophotometry. In addition, a non-invasive method of buccal cell brushing [43] was used to obtain DNA from premature infants, recipients of previous blood transfusions and infants with meconium ileus. Mutation screening of the entire coding sequences of the *CFTR *gene (including the 27 exons and exon/intron boundaries, intron 11 and 19, as well as the promoter region) was performed for each patient using a well-established single strand conformation polymorphism/heteroduplex (SSCP/HD) analysis. This was followed by direct sequencing of the variants, thus permitting rapid and sensitive detection of 97 – 98% of known and novel (newly identified) CF mutations, as previously described [34,44,45].

### Microbiology

Sputum and throat swabs were obtained at each follow-up visit and cultured for various bacterial species including *H. influenzae, S. aureus *and *P. aeruginosa *[46]. Sampling, transport, culture and identification of strains from respiratory secretions were performed according to standard procedures [46]. Sputum specimens were processed by the Institute of Microbiology, University of Berne, where they were inoculated on blood, chocolate and MacConkey agars [47]. Strains of *P. aeruginosa*, *Staphylococcus aureus *and *Haemophilus influenzae *were tested for antibiotic susceptibility by the Kirby-Bauer paper disk method.

### Data computation and statistical evaluation

In order to present individual values of lung function numerically and independent of gender, age and growth status, all lung function data were expressed as standard deviation score (SDS). The value obtained by z-transformation [48] indicates the number of standard deviations (SD) a CF-patient deviates from the gender- and age-specific regression equations for healthy subjects reported previously [38-40]. Repeated measurements of lung function data were first calculated as mean ± SEM values per year over age for synoptical presentation. Linear mixed-effect model (LMM) analysis was used to assess the relationship between each lung function parameter and age [16,49-52], (*i*) to obtain reliable estimates of individual changes over time of an outcome, and hence, to examine progression of each lung function parameter, and (*ii*) to study the role of potentially associated factors such as specific *CFTR *genotypes or bacterial colonization over the age range of 6–8 to 18 years. This technique is suited to analysis of the association between time and covariates from irregularly spaced serial data from individuals (i.e. repeated measurements), without being affected by missing data [49-52]. The various lung function parameters were modelled with age at observation as fixed effect, and a patient-specific intercept as random effect. A *t*-test assuming unequal variances was then performed to determine if regression slopes of the different lung function parameters in the whole sample differed from zero, and to test for differences of the regression slopes between groups. Holm's modification of the Bonferroni correction for multiple comparison was applied. The *p*-values significant at the 0.05 level after this correction are marked with an asterisk in the text and tables. Results with a significance level of p < 0.05 were considered statistically significant. Prism software (version 4.0, GraphPad Software, Inc., San Diego, USA) was used for graphical, and SPSS (version 11, SPSS Inc., Chicago, USA) for statistical analysis.

## Results

### Characteristics of the study population

The current Bernese Cystic Fibrosis Patient Data Registry contains data from a total of 190 CF patients who have been followed over the last 28 years. From this collective 152 (76.8%) fulfilled the inclusion criteria defined for the present study (Table 1). Fifteen patients have not yet reached the age of 6 years, and in 23 CF patients less than 4 annual lung function tests were available. Gender was approximately equally distributed. Within these 152 CF patients a total of 1460 lung function tests were performed, representing a median (range) of 10 (4 – 15) lung function tests per child, or 83 (29 – 116) lung function tests per year.

**Table 1:** Patient cohort, data base characteristics, distribution of *CFTR *mutations, and stratification into different types of bronchial infection in study patients with cystic fibrosis

**Patient **(from database*)			**Follow-up statistic **(from database*)			
	*n*	*%*	number of tests		age ranges covered	

all	152		total	1460	6 to 10 y	80%
- males	77	50.7	per child	10 (4–15)	11 to 15 y	71%
- females	75	49.3	per year	83(29–116)	16 to 20 y	39%

**CFTR mutation stratification**						

	*n*	*%*				

ΔF508(2)	86	56.6	**Miscellaneous**: numbers in brackets			
3905insT/ΔF	13	8.6	ΔF508 and1717-1G>A(4), W1282X(4), 2347delG(3), G524X(2), Q525X(2), N1303K(2), 621+1G>T(1),			
R553X/ΔF	10	6.6	2176insC(1), 394delTT(1), 4005+1G-A(1). 420del9(1), E585X(1), G126D(1), G85E(1), R347P(1), 1078delT(1);			
Miscellaneous	43	28.3	3905insT and1717-1G>A(1),K710X(1), M1101K(1), Q39X(1), P5L(1), R553X(1); R553X andR553X(1); G542X and T5(3), G542X(1); Q542X and3732delA(2); N1303K and2347delG(1), 2789+5G>A(1); 1199delG andR560S(1).			

**Stratification into different types of infection**						

	*n*	*%*				

free from any	6	3.9				
intermittend with various*	34	22.4	* *H. influenzae, S. aureus, St. maltofilia*			
*S. aureus*	19	12.5				
*P. aeruginosa*	36	23.7				
*P. aeruginosa *combined *S. aureus*	57	37.5				

*Actual number of patients in database: 198

Number of patient under age of 6 years: 13

Number of patients with follow-up data less than 4 annual lung function tests: 23

According to the frequencies in our population-specific *CFTR *genotype distribution the patients were stratified into 4 *CFTR*-specific groups (Table 1). Group 1 consisted of those with a homozygous ΔF508 mutation (ΔF508(2): n = 86, 56.6%). Group 2 included compound heterozygotes for the second most common mutation found in Switzerland, 3905insT and ΔF508 (3905insT/ΔF: n = 13, 8.6%). Compound heterozygotes for the nonsense mutation R553X and ΔF508 constituted group 3 with the third most common genotype (R553X/ΔF: n = 10 6.6%), whereas the fourth group comprised 43 miscellaneous genotypes (28.3%).

Stratification into different types of bronchial infection (Table 1) was performed by defining those free of any colonization (n = 6, 3.9%), those presenting with intermittent colonization with one or more positive cultures of either *H. influenzae*, *S. aureus, or St. maltofilia *(n = 34, 22.4%), those chronically infected with *S.aureus *(n = 19, 12.5%), those chronically infected by *P. aeruginosa *(n = 36, 23.7%), and those culture positive for both *P. aeruginosa *and *S. aureus *(n = 57, 37.5%).

### Progression of lung function over time

**Figure 1:**
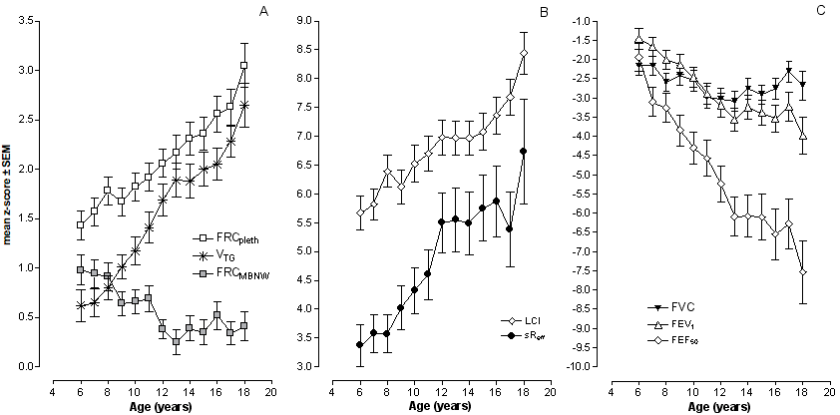
**Progression of lung function with age**. A) Changes assessed by repeated measurements of plethysmographic functional residual capacity (FRC_pleth_), functional residual capacity obtained by the multibreath nitrogen washout (FRC_MBNW_), and volume of trapped gas (V_TG_). V_TG _was calculated as the difference between FRC_pleth _and FRC_MBNW_. B) Changes of lung clearance index (LCI) as a measure of ventilation inhomogeneities and effective specific airway resistance (sR_eff_), as measure of airway narrowing. C) Changes of forced vital capacity (FVC), forced expired volume in one second (FEV1) and maximal expired flow at 50% FVC (FEF_50_) in relation to age. All parameters expressed as z-scores.

Figure 1 shows mean annual changes of static lung volume (panel A), changes in LCI, and sR_eff _(panel B) as estimates of intrapulmonary gas distribution and airway narrowing, and changes in flow volume curve derived indexes (panel C) in relation to patient age over an age range of 6 to 18 years. Values are presented as mean z-scores, equal to SDS ± SEM. FRC_pleth_, obtained by whole body-plethysmography, increased from 1.43 ± 0.15 SDS at age 6 y to 3.05 ± 0.22 SDS at the age of 18 y. Thus, while 38.7% of patients aged 6 to 8 yrs were found to have pulmonary hyperinflation (SDS-value > 2), the proportion of children continued to increase, with 67.0% observed to have hyperinflation at 18 y. Trapped gas volume also increased from 0.62 ± 0.16 at 6 y to 2.65 ± 0.22 at 18 y. The proportion of patients with V_TG _increased from 15% to 54% during this period. In contrast, FRC_MBNW _values obtained by multibreath washout decreased during this period from 0.98 ± 0.15 SDS at age 6 y to 0.41 ± 0,16 at 18 y.

Table 2 demonstrates the age related progression of all lung function parameters with the exception of FVC as assessed by LMM analysis and expressed as the changes occurring in mean regression slope for each index. Rates of progression were most significant for FEF_50 _(slope: -0.505, *p *< 0.0001), sR_eff _(slope: 0.381, *p *< 0.001), and LCI (slope: 0.281, *p *< 0.001). Less pronounced progression was also identified for pulmonary hyperinflation (FRC_pleth_: slope: 0.154, *p *< 0.0001),) and trapped gas (V_TG_: slope: 0.185, *p *< 0.0001).

**Table 2:** Progression with age (slope of regression) assessed by linear mixed-effect model analysis (LMM) in 152 patients with cystic fibrosis, evaluated over an age-range of 6 to 18 years.

	**Progression with age of lung function**	95% confidence interval		**Age as fixed effect**	
		lower	upper	*F-value*	*Significance*

					
**FRC_pleth_**	0.142	0.126	0.158	298.3	0.0001
**FRC_MBNW_**	-0.062	-0.081	-0.043	42.1	0.001
**LCI**	0.240	0.204	0.276	174.4	0.0001
**V_TG_**	0.180	0.160	0.200	320.9	0.0001
**sR_eff_**	0.373	0.319	0.427	182.1	0.0001
**FVC**	0.005	-0.023	0.033	0.1	n.s.
**FEV_1_**	-0.177	-0.206	-0.148	139.6	0.0001
**FEF_50_**	-0.474	-0.527	-0.420	304.0	0.0001

### Effect of early pulmonary hyperinflation and gas trapping on later functional outcome

**Figure 2:**
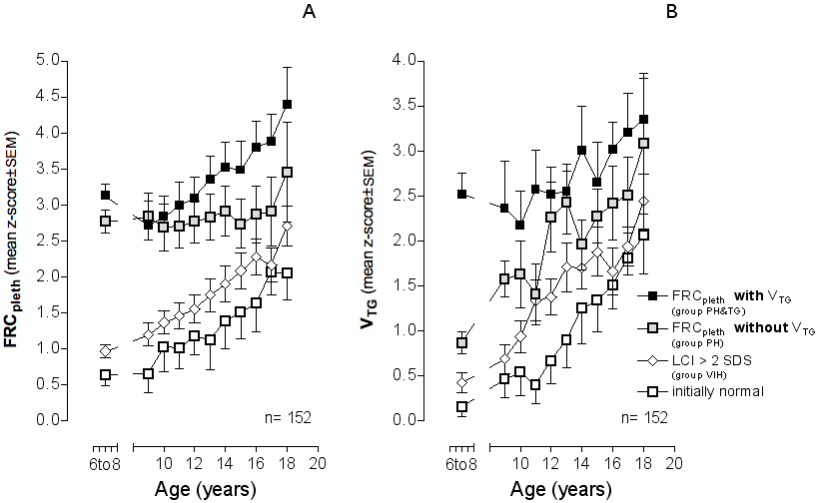
**Progression of FRC_pleth _over time in 152 patients with lung function stratified at age 6 to 8 years into 4 functional severity groups**. Group PH&TG (pulmonary hyperinflation and trapped gas): FRC_pleth _and V_TG _> 2SDS; group PH (pulmonary hyperinflation without trapped gas): FRC_pleth _> 2SDS; group VIH (ventilation inhomogeneities): LCI > 2SDS; group FN: functionally normal.

Figure 2 shows the progression of FRC_pleth _(A) and V_TG _(B) over time for each of the 4 functional groups, stratified according to age at entry (age 6 to 8 yrs). Children initially presenting with both pulmonary hyperinflation and trapped gas (group PH&TG) demonstrated highest values for both FRC_pleth _and V_TG_. These patients also showed consistently higher degrees of hyperinflation over time in comparison to those in whom pulmonary hyperinflation occurred in the absence of trapped gases (group PH). Furthermore, age related progression of disease was associated with development of similar degrees of gas trapping between functional groups as evidenced by the similar slopes of these parameters in Figure 2. Occurrence of ventilation inhomogeneities in the absence of hyperinflation was associated with both progression of both FRC_pleth _and trapped gas. Presence of initially normal lung function or early ventilation inhomogeneities still resulted in progressive elevation of both FRC_pleth _and V_TG _over time. However, CF patients with early severe functional deficits (groups PH and PH&TG) showed consistent differences (p < 0.001) from the other groups, which persisted throughout the entire duration of the study (i.e. demonstrated tracking).

### Relationship between lung function parameters and CFTR genotype

Associations between lung function and *CFTR *genotypes, progression of changes in specific functional indexes within *CFTR *genotype groups and comparisons between functional groups are given in Table 3. Potential associations were assessed by LMM analysis incorporating data from the 3 specific mutation groups (i.e. excluding the group comprising miscellaneous genotypes). Age and the 3 most frequent *CFTR *groups were taken as fixed effects and the patient-specific intercept as random effect. The most significant age related progression was identified within the 3905insT/ΔF mutation group for FEF_50 _(slope: -0.738) and sR_eff_(slope 0.549; panel **A**). The effect of mutation group was assessed by analyzing the position of the intercept through the ordinate. This value was found to be significantly higher for LCI (5.077), and FEV_1 _(4.542, panel **B**). With the exception of LCI, significant differences in the values for the regression slope were found between *CFTR *genotype and lung function indices. The strongest associations were observed for FEF_50 _(*F *= 14.255, *p *< 0.0001) and FEV_1 _(*F *= 13.066, *p *< 0.0001). FEF_50 _differentiated best between *CFTR *genotypes, if ΔF508(2) group was taken as the baseline value (Table 3, panel **C**). Children in whom the R553X/ΔF mutation was present demonstrated the lowest values for all lung function parameters at time of initial measurement (age 6 to 8 yrs). Those with the ΔF508(2) mutation had higher initial values. Maximum values for parameters obtained at initial measurement were observed in the 3905ins group.

**Table 3:** Progression with age of lung function within genetic groups stratified according frequency of *CFTR *mutations.

		***A***	**Progression with age of lung function**	***B***	**Comparison of progression between genetic groups**				**C**	Comparison of progressions within groups in relation to ΔF508(2) *		**D**	delta of **Power analysis **0.8
			*Slopes within groups*		Intercept at age 6 to 8 yrs mean comparison		Slope differences (age range 6 to 18 yrs)						

					*F-value*	*sign*.	*F-value*	*sign*.		*mean diff*.	*sign*.		

**FRC_pleth_**	ΔF508(2)		0.151		1.117	n.s.	3.979	0.008					
	3905insT/ΔF		0.215							1.057	0.048		0.09154
	R553X/ΔF		0.165							0.120	n.s.		0.09969
													
**LCI**	ΔF508(2)		0.247		5.077	0.002	0.491	n.s.					
	3905insT/ΔF		0.291							1.865	0.006		0.200854
	R553X/ΔF		0.278							0.307	n.s.		0.236018
													
**V_TG_**	ΔF508(2)		0.198		3.372	0.019	6.499	0.0001					
	3905insT/ΔF		0.233							1.036	0.011		0.113801
	R553X/ΔF		0.256							0.065	n.s.		0.128347
													
**sR_eff_**	ΔF508(2)		0.405		0.849	n.s.	10.043	0.0001					
	3905insT/ΔF		0.549							1.297	n.s.		0.305402
	R553X/ΔF		0.741							3.298	0.039		0.328812
													
**FEV_1_**	ΔF508(2)		-0.185		4.542	0.004	13.066	0.0001					
	3905insT/ΔF		-0.216							2.502	0.009		0.16809
	R553X/ΔF		-0.466							0.431	n.s.		0.18238
													
**FEF_50_**	ΔF508(2)		-0.439		1.774	n.s.	14.255	0.0001					
	3905insT/ΔF		-0.738							1.011	n.s.		0.30235
	R553X/ΔF		-1.029							2.952	0.002		0.32759
	Misc		-0.354										

* adjusted for multiple comisons according Bonferroni

### Relationship between lung function and different combinations of infection

**Figure 3:**
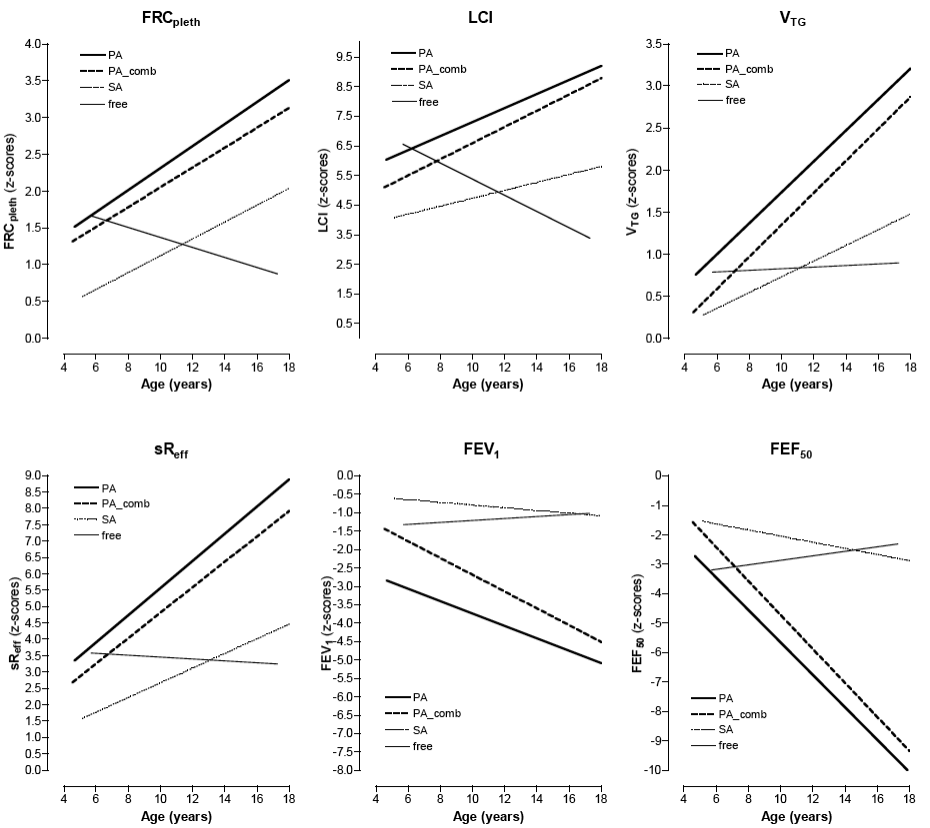
**Progression with age within 5 different types of colonization or infection respectively, depicted for each lung function parameter**. Slopes were calculated from the fixed values predicted according to group using linear mixed-effect model analysis. (PA: chronically infected by *P. aeruginosa*; PA_comb: chronically infected by *P. aeruginosa *and other bacteria; SA: chronically infected only by *S. aureus*; intermit.: intermittently colonized by several bacteria; free: free from any bacterial colonisations).

The impact of different types of infection on progression of lung function is shown in Figure 3. Age and the 4 most frequent types of infection were taken as fixed effects and the patient-specific intercept as random effect. Children with chronic *P. aeruginosa *infection (PA) showed the most rapid rate of progression when examined for all lung function parameters. Within this group progressive changes in parameter values were most rapid for FEF_50 _(slope: -0.582) and sR_eff _(slope: 0.480). Group effects, *i.e*. initial already high intercept were detected for FEV_1 _(19.214), and LCI (7.345,). Significant relationships were identified between infection type and progression of lung function indexes, with strongest associations observed for FEF_50 _(*F *= 7.994, *p *< 0.0001) and FRC_pleth _(*F *= 6.020, *p *< 0.0001). Of all the groups in which infection was present, those with chronic *S. aureus *infection (*SA*) showed the least aggressive rate of progression of functional index values. Interestingly, although not significant but as tendency observed for each lung function parameter, *P. aeruginosa *combined with other infection (*PA_*comb) presented with more progression than *P. aeruginosa *infection (*PA*) alone. LCI proved to be the index most sensitive for differentiating between infection types (*p *< 0.0001) when the group free of any colonization or infection was taken as baseline.

## Discussion

This study demonstrates that progression of pulmonary hyperinflation and the presence of trapped gas are important mechanical features of disease evolution in patients with cystic fibrosis. Data analyzed from a cohort of 152 CF patients, revealed the presence of pulmonary hyperinflation in more than one third (37.5%) of cases as early as age 6 to 8 years. In half of these (18.4%), pulmonary hyperinflation was associated with trapped gas. Both functional abnormalities deteriorated with age (Figure 2). Ventilation inhomogeneities have been previously shown to represent the earliest and most rapidly progressive functional abnormality in CF [16]. The current findings suggest that ventilation inhomogeneities are accompanied by steadily increasing hyperinflation, gas trapping, airway obstruction, and flow limitation. All patients identified as having increased V_TG _also demonstrated increased values for LCI. Rate of progression of functional abnormality was most rapid within a subgroup of patients within whom both pulmonary hyperinflation and trapped gas were present (Figure 2).

The relationships between pulmonary hyperinflation and gas trapping and deterioration of lung function in CF are presented here using longitudinal data. Previous investigations have demonstrated that pulmonary hyperinflation influences lung mechanics in terms of increased work of breathing, greater severity of breathlessness, impaired respiratory muscle function [53-58] and increased energy expenditure and oxygen consumption [55,56,59]. Recognition of functional deterioration is therefore critical to the ongoing management of patients with CF. Trapped gas occurs as a consequence of absent communicative pathways between small and large airways, thus reducing the alveolar surface area available for gas exchange [37,42,60]. Pulmonary hyperinflation and the development of trapped gas are closely associated with different types of chronic bronchial infection, especially *P. aeruginosa *(Figure 3). Of even greater interest, our results suggest that the *CFTR *genotype plays an important role in determining the longitudinal functional progression of lung disease in CF (Table 3).

Finally, this work in CF patients provides further confirmatory evidence for progressive tracking of lung function abnormalities already observed in other chronic respiratory illnesses such as bronchial asthma [27] and chronic lung disease of infancy [25]. Ranganathan et al. demonstrated tracking between parameters of airway function and growth in infants and young children [25]. Children with CF and better initial FEV_1 _have a slower rate of decline in lung function than those in whom initial FEV_1 _was already very low [61]. The authors concluded that young children with good pulmonary function and intercurrent pulmonary illness need not be treated as aggressively as children with documented lower FEV_1_. Our own data support this finding, since children with evidence of severe disease early in life experienced more aggressive functional deterioration over the course of the study period.

### Changes of lung volume during disease progression in CF

Elevation of FRC represents an almost universal accompaniment of significant intrathoracic airway obstruction. Elevated end-expiratory level in patients with severe lung disease is achieved by a strategy adopted to increase expiratory flow, especially during exercise. Patients thus meet their ventilatory requirements at rest by increasing breathing frequency rather than tidal volume in order to minimize the increase of resistive work associated with thoracic wall excursion. However, pulmonary hyperinflation affects respiratory muscle function [62]. Elevation of end-expiratory level above relaxation volume places an extra load on the inspiratory musculature at end-expiration, whereby an additional "threshold" load related to the elastic recoil of the respiratory system must be overcome prior to commencement of inspiratory flow. Hyperinflation, together with loss of static recoil occurring in relation to airflow limitation results in altered respiratory muscle function [55,56]. There continues to be only limited understanding of how respiratory muscle function is altered in patients with hyperinflation. Animal experiments indicate that hyperinflation is detrimental to the functional effectiveness of the diaphragm, but may provide mechanical benefit to the parasternal intercostals [63]. Patients with severe hyperinflation demonstrate more pronounced diaphragmatic shortening than intercostal and accessory muscle shortening [64]. These patients also exhibit clear signs of reduced diaphragmatic functional capacity which correlates with the degree of airflow obstruction [64,65]. Studies of interactions between pulmonary hyperinflation and inspiratory muscle function have highlighted the reduced muscular efficiency and predisposition to fatigue occurring in CF [57].

Preferential impairment of the peripheral skeletal musculature is frequently seen in patients with CF. Typically, respiratory muscle strength is preserved while the quadriceps is weakened [56]. This observation is consistent with the diaphragm benefiting from a continuous training stimulus secondary to increased inspiratory impedance. Therefore, hyperinflation does not impair force generation of the diaphragm to the same extent in cystic fibrosis as may occur in other chronic respiratory diseases. Normal inspiratory muscle strength is commonly observed in CF and diaphragmatic adaptation in this condition may extend beyond that usually observed in adult COPD, where maximal diaphragmatic strength is lower than in normal individuals. The relative contribution of inspiratory musculature to overall respiratory muscle weakness in CF patients has also been assessed [66]. Major determinants of inspiratory muscle weakness include muscle mass, hyperinflation, gas trapping and to a lesser extent nutritional status [56]. Thus, gradual deterioration in pulmonary function, together with an associated increase in work of breathing and inspiratory muscle weakness all play important roles in the development of ventilatory failure [53,54,57,58].

### The role of the development of trapped gas

Identification of early pulmonary hyperinflation associated with trapped gas as the most severe functional group during childhood and adolescence may represent an early warning signal. Early gas trapping in this setting appears to progress to persistent and ongoing disruption of lung mechanics, leading to impairment of gas exchange [60,67-71], increased energy expenditure [59,72], and disruption of normal chest wall motion and exercise performance [73]. Together with chronic infection, poor energy intake and a catabolic state [55,56,59], further deterioration of respiratory muscle function occurs [55,56]. A reduction in trapped gas volume in association with improved maximal working capacity is observed in CF patients following long-term chest physiotherapy [74,75]. In normal subjects, expiration to residual volume results in a degree of small airway closure, some atelectasis of dependent lung regions and gas trapping [76]. The extent to which this occurs is related to age [77], as highlighted recently by Milic-Emili [78]. Closure of small airways results in discontinuity of expiratory airflow. Healthy subjects maintain FRC above a critical volume at which airway closure occurs (closing volume) and therefore demonstrate no gas trapping [79]. In young children, closing volume and FRC approximate each other closely [79], thus further potentiating gas trapping. In the presence of disease, and particularly in CF, impaired airway clearance mechanisms and inflammatory changes associated with chronic infection disrupt small airway patency, leading to discontinuity and limitation of expiratory airflow. Loss of static recoil associated with pulmonary hyperinflation may further aggravate this process [80] and increase the potential for gas trapping[79]. We have recently shown that an index of ventilation inhomogeneity, the LCI, progresses with age in patients with CF, particularly after the onset of chronic *P. aeruginosa *infection [16] and allergic bronchopulmonary aspergillosis (ABPA) [41]. Whilst an increase in gas trapping results in a concordant rise in ventilation inhomogeneities, the degree to which airway closure occurs will also depend upon the age-dependant relationship between FRC and closing volume and on airway resistance [77,79,81-83]. Since the separation between FRC and closing volume is likely to be reduced at younger ages, the appearance of severe ventilation inhomogeneities and pulmonary hyperinflation in this age group may predispose towards early gas trapping and earlier onset of age related progression of lung function deterioration (tracking). Early identification of gas trapping may therefore be critical to instituting therapeutic measures aimed at retarding or reversing this situation.

### Relationship between CFTR genotypes and progression of hyperinflation and gas trapping

A relationship between *CFTR *genotype and severity of pulmonary disease in CF has proven difficult to establish. Nevertheless, the variability of pulmonary function at time of diagnosis in infants [21] and children [84-86] has been found to be partially related to the genotype. In comparison to the inframe homozygotes ΔF508(2) and nonsense R553X/ΔF compound heterozygotes, patients carrying one frameshift mutation 3905insT have a poorer prognosis with respect to the onset of pulmonary disease, progression of lung function, and mortality [87]. Schaedel et al. used FEV_1 _% normal predicted to demonstrate a slower rate of decline in patients with missense mutations compared with ΔF508(2) homozygotes [86], and Cory et al. used LMM analysis to show a slower rate of pulmonary function decline in some patients with non-ΔF508 mutations [51]. We undertook a similar statistical approach to evaluate potential associations between repeated lung function measurements and the most frequent *CFTR *genotypes in Switzerland, ΔF508(2), 3905insT/ΔF and R553X/ΔF. Significant differences in lung function indices were identified between the 3905insT/ΔF compound heterozygote and ΔF508(2) homozygote mutation groups for FRC_pleth_, LCI and V_TG_, as well as between R553X/ΔF compound heterozygotes and ΔF508(2) homozygotes for sReff, and FEF_50 _(Table 3). Confirming previous findings [15,21,51,88,89], our data demonstrate that the 3905insT mutation is associated with severe lung disease, manifesting early in life [21], whereas the R553X/ΔF mutation seems to provide milder pulmonary involvement during the first 5 to 6 yrs of life, thereafter however, to be exposed to a much more pronounced progression compared with both the ΔF508 and the 3905insT/ΔF. In addition, regressions of fixed predicted values obtained by the LMM analysis indicate significantly higher rates of progression of small airway disease in patients within the 3905insT/ΔF group compared to ΔF508(2) homozygotes. The effect is even more pronounced for the R553X/ΔF group. Thus, we conclude that *CFTR *genotypes clearly act as an important determinant of disease progression and hence outcome, when lung function parameters are interpreted in terms of variance-based data (*z*-scores), and in relation to gender- and age-specific regression equations [38-40].

### The role of environmental factors

In the present study patients chronically colonized with *P. aeruginosa *showed a significantly worse disease course (Figure 3) compared to other types of infection. This was especially the case in relation to ventilation inhomogeneities. This finding is consistent with observations of Wilmott et al. showing a strong association between *P. aeruginosa *status and mortality [90], and the associations between FEV_1 _and *P. aeruginosa *infection found by Kerem et al [91]. More noteworthy, is the finding that in addition to age of onset of chronic *P. aeruginosa *infection, colonization status at the time of lung function evaluation is important.

### Methodological limitations

The current study represents a very large patient cohort, many of whom were followed up over a long time period. An important limitation of these types of data resides in the ability to obtain repeated measurements of lung function annually, over a substantial range of time. However, we obtained serial annual measurements over a 10-year period in at least 50% of the children. Linear mixed-effects model analysis provides an ideal statistical method for the interpretation of repeated measurement data such as these, particularly when repeated testing results in small proportions of incomplete data. A second potential influence on the results of this type of analysis involves the use of subgroups, since the relatively small number of patients within each group may result in relatively high levels of variability. The adequacy of stratification within subgroups is critical to the interpretation of differences observed in outcome measures between each subgroup. Whilst stratification of bronchial infection subgroups was adequate, stratification of specific *CFTR *genotypes required a frequency based approach to search for significant associations. This finding may be intriguing for some research groups where genotype-phenotype associations with pulmonary involvement have not been able to be identified in CF. Whilst well-established associations between pancreatic insufficiency and genotype are recognised based on a clear "on-off" selection, studies investigating lung function indexes and genotype associations may need to consider several functional parameters in order to clearly identify those likely to be affected by mutational changes. Our results suggest that parameters representing pulmonary hyperinflation, ventilation inhomogeneities, gas trapping and airway narrowing are required to be considered in addition to those quantifying the degree of bronchial obstruction. Moreover, lung function data must be presented in a form independent of gender and growth status (z-transformation). The current study presents longitudinal data and expresses changes in SDS in terms of cross-sectional reference equations [16]. The volume of trapped gas was calculated as the difference between FRC measured plethysmographically (FRC_pleth_) and by the FRC obtained by gas washout (FRC_MBNW_). It must be borne in mind that the difference between these two values provides an index that correlates with trapped gas, but is not equal to trapped gas. In the presence of significant airway obstruction, FRC is overestimated by plethysmography. However, we used a non-panting method that reduces this artifact by allowing greater time for equilibration between alveolar and mouth pressure. Finally, genotype-phenotype association studies investigating pulmonary involvement in CF are complex and require determination of factors such as available lung function parameters and duration of life span during which data are acquired. A specific question is how the genetic background should be categorized. Possible categorizations might include ΔF508 versus non-ΔF508, or in terms of molecular mechanisms and consequences observed at the gene or protein level. It has to be kept in mind, that our cohort study represents primarily the *CFTR *genotype distribution for Switzerland, where a substantial proportion of patients carry the severe CF mutation (3905insT/ΔF), which is associated with the highest mortality rate [21,37,44,88].

## Summary

This study demonstrates that pulmonary hyperinflation and development of trapped gas represent major functional features of disease progression in children with CF. Children with severe pulmonary hyperinflation and gas trapping at age 6–8 y have the most significant rate of disease progression over time. As has been reported previously in childhood asthma, tracking of lung function abnormalities in CF commences early in life, and is considerably influenced by the *CFTR *genotype. Furthermore, the present study shows that FRC_pleth_, and hence the degree of pulmonary hyperinflation best differentiate between different types of bronchial infection. Chronic *P. aeruginosa *infection appears to be the most important infective contributor to disease progression. The observed associations between *CFTR *genotypes and lung function characteristics, as well as the associations between different types of bronchial infection with pulmonary hyperinflation stress the need to include a range of tests when assessing these patients, rather than relying simply on spirometry. Early assessment of airway obstruction, pulmonary hyperinflation and gas trapping in addition to ventilation inhomogeneities and in conjunction with *CFTR *genotyping provides a means for monitoring functional progression in CF disease.

## Abbreviations

BTPS: body temperature and pressure saturated

CF: Cystic Fibrosis

*CFTR*: Cystic Fibrosis Transmembrane conductance Regulator

DNA: Deoxyribonucleid Acid

FEV1: Forced Expiratory Volume in One second

FEF_50_: Forced expiratory flow at 50 percent FVC

FRC_pleth_: Functional residual capacity (plethysmographically determined)

FRC_MBNW_: Functional residual capacity (determined by MBNW)

FVC: Forced Vital Capacity

LCI: Lung clearance index

LMM: Linear mixed model

MBNW: Multibreath nitrogen washout

PA: *Pseudomonas aeruginosa*

SA: *Staphylococcus aureus*

SDS: Standard deviation score

SEM: Stardard error of the mean

sR_eff_: specific effective airway resistance

SSCP/HD: single strand confirmation polymorphism/heteroduplex

TLC: Total Lung Capacity

V_TG_: volume of trapped gas

## Competing interests

The author(s) declare that they have no competing interests.

## Authors' contributions

RK designed, coordinated and conceived the study.

DB took part in the interpretation of data and manuscript revision.

RA participated in the data collection, interpretation of data and manuscript revision

UF took part in the interpretation of data and revised the draft.

SG performed the CF mutation screening, took part in the interpretation of data (especially genetics) and revising.

All authors read and approved the final manuscript.

## Funding

The study was supported by grants of the Swiss National Research Foundation (SNF 3200-040681.94_SG, 32-040562.95_RK, 3200-055697.98 _SG, 32-066767.02_SG) and the Foundation Telethon Action Switzerland. Dr. Baldwin was a fellow in the ERS Long Term Research Program.
